# A Rare Case of Metastases from a High-grade Astrocytoma to the Pleura, Bones, and Liver within Six Months of Diagnosis

**DOI:** 10.7759/cureus.3234

**Published:** 2018-08-30

**Authors:** Jennifer Dotson, Ala Nijim, Krista L Denning, Yousef Shweihat, Yehuda Lebowicz

**Affiliations:** 1 Joan C. Edwards School of Medicine, Marshall University, Huntington, USA; 2 Pulmonology, Marshall University, Joan C. Edwards School of Medicine, Huntington, USA; 3 Pathology, Marshall University, Joan C. Edwards School of Medicine, Huntington, USA; 4 Internal Medicine, Marshall University, Joan C. Edwards School of Medicine, Huntington, USA; 5 Hematology and Oncology, Marshall University, Joan C. Edwards School of Medicine, Huntington, USA

**Keywords:** astrocytoma, metastases, pleura

## Abstract

High grade astrocytomas such as anaplastic astrocytoma and glioblastoma multiforme are aggressive central nervous system malignancies with a poor prognosis. Due to shortened survival times, their devastating effects are usually localized intracranially and rarely metastasize outside of the central nervous system. When metastases occur, they usually present in patients with longer survival times and they typically coincide with a primary site recurrence. We present a rare case of metastases from a high-grade astrocytoma/glioblastoma to the pleura, bones and liver within six months of diagnosis, without primary site recurrence.

## Introduction

High-grade astrocytomas or glioblastomas are aggressive primary central nervous system (CNS) tumors with overall poor prognosis. They are typically divided into three categories: anaplastic astrocytomas, anaplastic oligodendroglioma and glioblastoma multiforme and are graded from III-IV [1]. Glioblastomas are four times more common than anaplastic astrocytomas, with incidences of around two to three per 10,000 people in the United States [2]. Glioblastomas fare worse in prognosis with median overall survival of only around 12 months, compared to anaplastic astrocytomas with median survival of around two to three years [3]. These tumors usually remain confined to a localized area and rarely metastasize outside of the CNS [3]. However, extracranial metastases have been surmised in the literature to occur in 0.44-2% of these patients [4-6]. Pleuropulmonary metastasis from an intracranial glioblastoma is exceedingly rare, often due to the shortened survival of these patients. Additionally, metastases usually occur in the setting of primary site reoccurrence or progression. We describe a case of previously diagnosed high-grade astrocytoma with presentation of metastases only six months after initial diagnosis with extra-CNS metastases to the pleura, liver and bone without primary site recurrence.

## Case presentation

A 56-year-old Caucasian male with past medical history of a high-grade astrocytoma of the right temporal lobe presented to the oncology clinic with dyspnea and palpitations for several weeks. He had been diagnosed with high-grade astrocytoma six months prior, which was treated with surgical resection and was followed by concurrent chemotherapy and radiation for six weeks with temozolomide with subsequent maintenance temozolomide. Pathology from his original brain tumor noted mixed features of an anaplastic pleomorphic xanthoastrocytoma (PXA) with atypical features versus glioblastoma. The pathologist described an astrocytic neoplasm composed of cells with variable polymorphism, brisk mitotic activity including atypical forms, and necrosis were present. Immunohistochemistry (IHC) stains were positive for glial fibrillary acidic protein (GFAP), oligodendroglial lineage 2 (OLIG2) and cellular differentiation 34 marker (CD34). The tumor did not harbor isocitrate dehydrogenase 1 (IDH-1), methylguanine-DNA methyltransferase (MGMT) or B-raf proto-oncogene serine/threonine kinase (BRAF) mutations.

**Figure 1:**
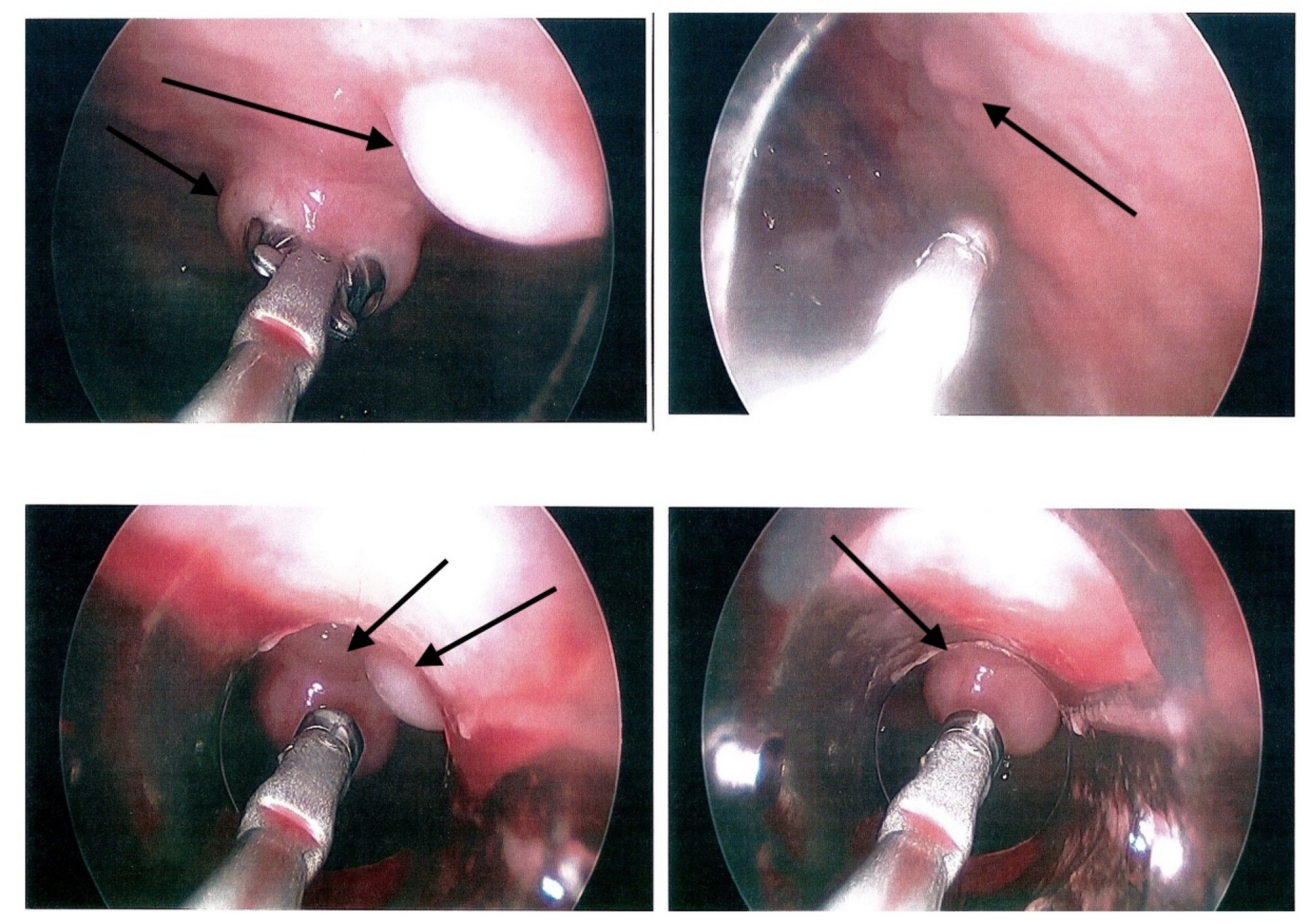
Photographs of the pleural space obtained during thoracoscopy show numerous nodules studding the pleura (indicated by arrows), suggestive of metastatic disease.

**Figure 2:**
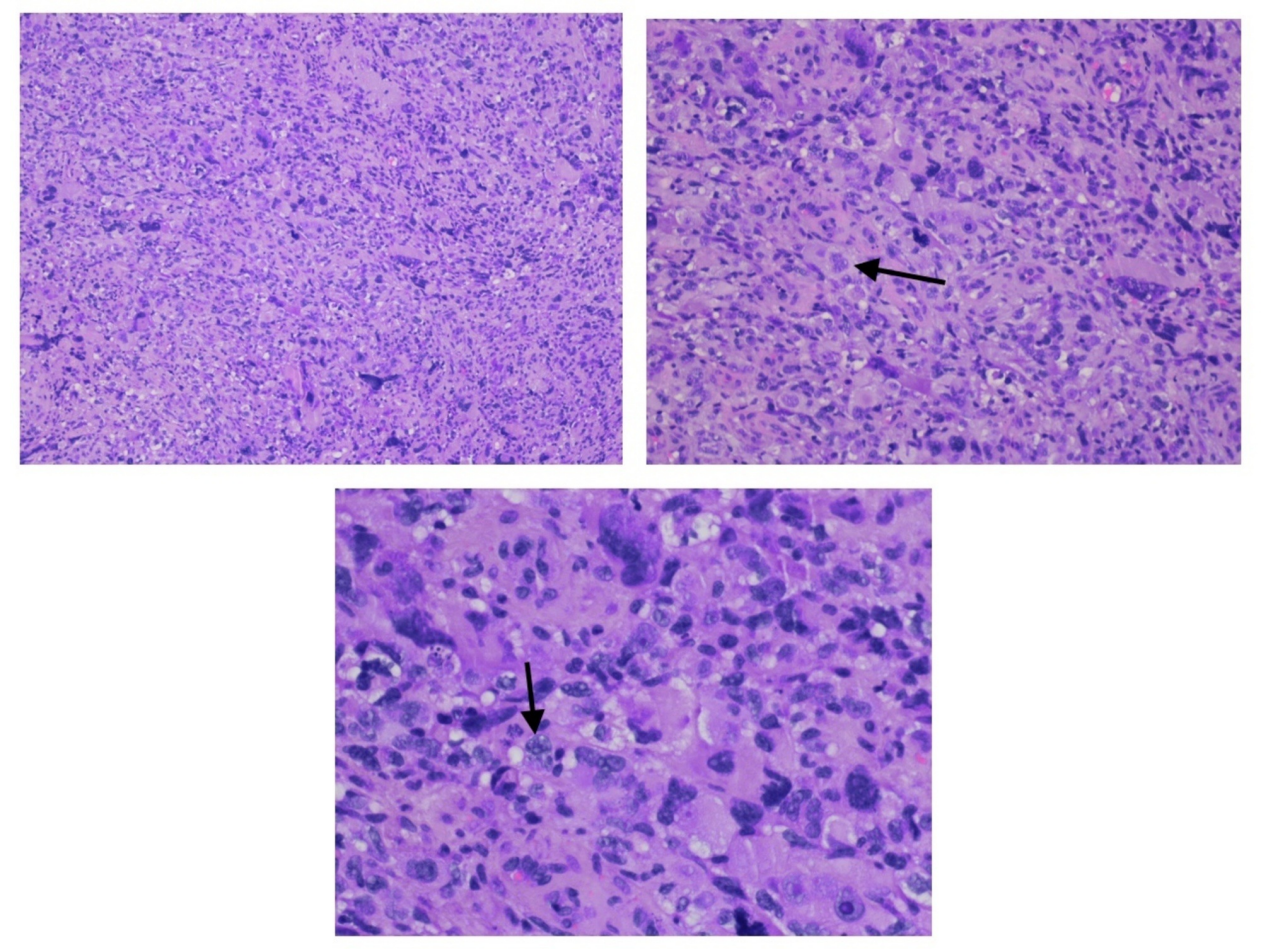
Histologic findings of the pleural biopsy show pleomorphic astrocytic tumor cells (notable for vacuolization of the neoplastic cells and indicated by the arrows) compatible with a pleomorphic high-grade astrocytoma (hematoxylin-eosin, 100× 200× and 400× original magnification).

Upon presentation to our clinic, the patient was still receiving maintenance therapy with temozolomide 200 mg/m^2^ on days one through five every 28 days. On physical exam, he was found to be tachycardic with a heart rate in the 150s with an irregularly irregular rhythm. On physical exam, he was noted to have decreased breath sounds to auscultation on the right and dullness to percussion in the mid to lower right hemithorax. He was admitted to the hospital for further evaluation. Computed tomography (CT) of the chest showed a pulmonary embolus in the left pulmonary artery in addition to a large pleural effusion on the right with pleural thickening and a nodular appearance. There was a new hypodense lesion in the superior part of the liver measuring 2.7 x 2.3 cm with multiple lytic bone lesions on CT of the abdomen. A bone scan confirmed the metastatic nature of bone lesions. Magnetic resonance imaging (MRI) of the brain showed abnormal enhancement in the middle cranial fossa, which was thought to be related to prior treatment. Anti-coagulation with heparin was initiated for treatment of the pulmonary embolus. Thoracentesis revealed the effusion to be exudative and cytology was significant for atypical cells. Thoracoscopy was performed for pleural biopsy and on examination, classic pleural studding was noted (Figure 1). Pleural biopsies were obtained using rigid optical forceps. Biopsy report showed malignant cells with initial IHC stains negative for calretinin, carcinoembryonic antigen (CEA), cytokeratin 5/6 (CK 5/6), pankeratin, thyroid transcription factor 1 (TTF-1) and Wilms tumor protein (WT-1), as is usually done to rule out lung carcinoma or mesothelioma. Because of marked pleomorphism in the sample which is normally seen in glioblastoma or high grade astrocytomas, glial fibrillary acidic protein (GFAP) by IHC was checked and found to be positive. Additionally, S100 (acidic protein, which is a common marker for neural tissue and melanoma) by IHC was also positive (Figure 2). Coupled together, these findings were consistent with metastasis from his known diagnosis of high grade astrocytoma/glioblastoma. The patient had placement of a Pleurx^TM ^catheter and was discharged home on anticoagulation therapy. Upon discharge and finalization of pathology report, the patient was offered chemotherapy with bevacizumab, but the patient opted to be placed in hospice and subsequently died a few weeks later.

## Discussion

Malignant gliomas are some of the most common brain tumors and generally have a poor prognosis [6]. Treatment includes a combination of surgical resection, radiation and cytotoxic chemotherapy. These gliomas are graded from I to IV and this is based on various histopathologic features such as atypia, necrosis and mitoses. Grade I tumors are considered benign while higher grades such as III and IV are considered malignant with a worse prognosis. These tumors are subcategorized into three different types: anaplastic astrocytoma (AA), anaplastic oligodendroglioma (AO) and glioblastoma multiforme (GBM). Anaplastic astrocytomas are grade III tumors which histologically have anaplasia and mitotic activity. Grade IV tumors, or glioblastomas, are histologically different in that they typically have microvascular proliferation and/or necrosis on pathologic specimens [1]. Of note, our patient was noted to have a high-grade astrocytoma, which was thought to be either high grade glioblastoma or pleomorphic xanthoastrocytoma (PXA) with atypical features [7]. PXAs are rare, accounting for less than 1% of gliomas, and are typically described as superficial astrocytic neoplasms, with histologic features of marked cellular pleomorphism and frequent xanthomatous change [8]. They are typically recognized as grade II gliomas but there are instances of aggressive variants with anaplastic changes and reports of transformation to glioblastomas [8,9]. Malignant gliomas in general remain localized to the central nervous system and do not typically metastasize outside of the brain, likely related to their shortened survival times. Metastases are exceedingly rare, initially estimated at 0.44% but has steadily increased to 2% of all GBM cases in recent years [4-6]. The median time for appearance of metastases is around two years from time of diagnosis [6]. Additionally, unlike our case, metastases usually coincide with recurrence or progression of the primary tumor site [10]. One review of the literature found 128 cases of metastatic GBM prior to 2008, which noted that the most common sites of spread occurred in the pleura (60%), regional lymph nodes (51%), bones (31%) and liver (25%) [5]. The increasing incidence of metastases outside of the central nervous system may potentially be explained by multiple factors, including the improvement in our imaging and diagnostic abilities to detect metastases in addition to newer treatment modalities and the improved overall survival of patients, which may take time for the metastatic cells to grow.

There are many hypotheses that attempt to explain why this particular type of tumor usually does not spread beyond the brain parenchyma. One hypothesis notes that the rarity of metastases may be explained both by shortened overall survival in addition to the peculiarity of the brain lymphatic system [4,11]. The blood-brain barrier is theorized to prevent migration of malignant cells outside of the CNS. However, malignant gliomas can utilize several mechanisms for metastases, including hematogenous spread via vessels of the primary tumor, hematogenous spread from invasion of the dural veins, hematogenous and lymphogenous spread by infiltrating the skull and extracranial soft tissues, spread via cerebrospinal fluid, and lastly, spread by ventricular shunting (ventriculoatrial or ventriculo-pleural shunts) [5]. With the improvement in treatment modalities over recent years, GBM cells may have more time to escape the CNS via shedding to lymphatic and hematological systems [11], though extracranial spread could also occur by contamination of blood during surgical tumor resection [12]. Huang et al. reported that about 96% of GBM patients with extracranial metastases previously underwent neurosurgical procedures [13]. Genetic derangements and molecular structures may also be a part in determining which patients will likely develop extracranial metastases [6]. Anti-angiogenic therapy has been reported as having a possible association with development of extracranial metastases [11].

## Conclusions

In conclusion, extracranial metastasis of high grade astrocytomas is very rare. Lung, lymph nodes, and bone are among the most commonly affected sites. It may or may not occur with a recurrence at the primary tumor site. The radiologic and clinical findings are nonspecific and it can mimic any other metastatic disease. Time to onset of metastases usually occurs in those patients with longer survivals. A high degree of suspicion is needed for early diagnosis and management of patients with this disease in order to hasten diagnosis, management and ultimately, survival.

## References

[1] Louis DN, Ohgaki H, Wiestler OD (2007). The 2007 WHO classification of tumours of the central nervous system. Acta Neuropathol.

[2] Hamilton J, Rapp M, Schneiderhan TM (2014). Glioblastoma multiforme metastasis outside the CNS: three case reports and possible mechanisms of escape. J Clin Oncol.

[3] Behin A, Hoang-Xuan K, Carpentier AF, Delattre J (2003). Primary brain tumours in adults. Lancet.

[4] Smith DR, Hardman JM, Earle KM (1969). Metastasizing neuroectodermal tumors of the central nervous system. J Neurosurg.

[5] Piccirilli M, Brunetto G, Rocchi G, Giangaspero F, Salvati M (2008). Extra central nervous system metastases from cerebral glioblastoma multiforme in elderly patients. Clinico-pathological remarks on our series of seven cases and critical review of the literature. Tumori.

[6] Beauchesne P (2011). Extra-neural metastases of malignant gliomas: myth or reality?. Cancers (Basel).

[7] Binesh F, Akhavan A, Navabii H (2012). Pleomorphic xanthoastrocytoma with malignant transformation and multiple recurrences in an Iranian girl. BMJ Case Rep.

[8] Burger PC, Scheithauer BW (2007). Tumors of the central nervous system. AFIP Atlas of Tumor Pathology, Series 4.

[9] Koga T, Morita A, Maruyama K (2009). Long-term control of disseminated pleomorphic xanthoastrocytoma with anaplastic features by means of stereotactic irradiation. Neuro Onc.

[10] Kim W, Yoo H, Shin SH, Gwak HS, Lee SH (2014). Extraneural metastases of glioblastoma without simultaneous central nervous system recurrence. Brain Tumor Res Treat.

[11] Elena A, Melina C, Raffaele N (2016). Extraneural metastases in glioblastoma patients: two cases with YKL-40-positive glioblastomas and a meta-analysis of the literature. Neurosurg Rev.

[12] Kalokhe G, Grimm SA, Chandler JP, Helenowski I, Rademaker A, Raizer JJ (2012). Metastatic glioblastoma: case presentations and a review of the literature. J Neurooncol.

[13] Huang P, Allam A, Taghian A, Freeman J, Duffy M, Suit HD (1995). Growth and metastatic behavior of five human glioblastomas compared with nine other histological types of human tumor xenografts in SCID mice. J Neurosurg.

